# Allyl nonanoate as a novel bile-derived biomarker in metabolic dysfunction-associated steatotic liver disease

**DOI:** 10.3389/fendo.2025.1707492

**Published:** 2025-10-28

**Authors:** Soo Hyeon Lee, Jonghwan Kim, Sung Ryol Lee, Bum Soo Lee, Ki Hyun Kim, Dae Won Jun, Chung Sub Kim, Kyung A Kang

**Affiliations:** ^1^ Department of Translational Medicine, Graduate School of Biomedical Science and Engineering, Hanyang University, Seoul, Republic of Korea; ^2^ Department of Biopharmaceutical Convergence, Sungkyunkwan University, Suwon, Republic of Korea; ^3^ Department of Surgery, Kangbuk Samsung Hospital, Sungkyunkwan University School of Medicine, Seoul, Republic of Korea; ^4^ School of Pharmacy, Sungkyunkwan University, Suwon, Republic of Korea; ^5^ Department of Gastroenterology, Hanyang University College of Medicine, Seoul, Republic of Korea; ^6^ Department of Radiology, Samsung Medical Center, Sungkyunkwan University School of Medicine, Seoul, Republic of Korea

**Keywords:** bile, hepatic fibrosis, metabolomics, metabolites, metabolic dysfunction-associated steatotic liver disease, MASLD

## Abstract

**Background:**

We analyzed metabolites directly obtained from gallbladder bile, providing novel insights and identifying a potential antifibrotic target in metabolic dysfunction-associated steatotic liver disease (MASLD).

**Methods:**

We conducted metabolomic profiling of gallbladder bile samples collected during cholecystectomy from 68 individuals, including 19 healthy controls and 49 MASLD patients with varying degrees of hepatic fibrosis. We used mass spectrometry-based metabolomics to identify fibrosis-associated metabolites. Functional validation included *in vitro* experiments in colon epithelial cells (HT-29), RNA sequencing, gene set enrichment analysis (GSEA), and a comprehensive G protein-coupled receptor (GPCR) screening assay.

**Results:**

A total of 68 subjects were classified into healthy controls (n = 19), fibrosis stage 0–1 (n = 35), and fibrosis stage 2–3 (n = 14). Bile metabolomic profiling revealed distinct clustering among the groups, with eight metabolites showing a stepwise increase with fibrosis severity. Using the Human Metabolome Database, eight fibrosis-associated metabolites (M1–M8) were identified, among which M5 (*m/z* 199.1693) was confirmed as allyl nonanoate. Allyl nonanoate levels were significantly higher in patients with hepatic fibrosis and increased progressively across fibrosis stages. Transcriptomic analysis demonstrated the downregulation of cell cycle-related pathways, including the G2/M checkpoint. GPCR screening identified GPR119 as the most responsive receptor, exhibiting the highest agonist activity (21.8%). The overexpression of GPR119 in HT-29 cells enhanced allyl nonanoate-induced GLP-1 expression.

**Conclusions:**

Bile metabolomic profiling in MASLD revealed distinct clustering patterns, with several fibrosis-associated metabolites demonstrating a stepwise increase with hepatic fibrosis.

## Introduction

Metabolic dysfunction-associated steatotic liver disease (MASLD) is the most common chronic liver disease with increasing prevalence on a global scale (1, 2). Despite its high prevalence and severity, there is no effective drug for MASLD treatment. It is probably because the pathophysiology of MASLD has not been clearly elucidated yet.

The pathogenesis of MASLD is multifactorial (3, 4). Bile acids contribute to disease progression by regulating fat absorption, lipid and glucose metabolism, and liver inflammation (5). Primary bile acids are synthesized in the liver from cholesterol, conjugated with glycine or taurine, and stored in the gallbladder before being secreted into the intestine. There, they are converted by gut microbiota into secondary bile acids. These bile acids act as signaling molecules via receptors such as the farnesoid X receptor and Takeda G protein-coupled receptor 5 (6, 7).

Recent studies have reported altered bile acid profiles, including changes in cholic acid, deoxycholic acid, and taurine-conjugated bile acids, in the serum and feces of metabolic dysfunction-associated steatohepatitis (MASH) patients (8, 9). However, because of complex multi-step modifications, serum and fecal bile acid profiles primarily reflect post-hepatic or microbiota-modified bile acid derivatives and may not capture liver-specific bile metabolic states. In contrast, bile samples directly collected from the gallbladder likely represent the liver’s immediate output and provide a unique opportunity to study intrahepatic bile metabolite dynamics relevant to MASLD pathogenesis. Despite this potential, studies using gallbladder bile remain scarce, likely due to the challenges in obtaining such samples.

Metabolomics can be used to identify biomarkers in various diseases, including MASLD (10). There have been several studies aimed at identifying the metabolites responsible for liver injury in MASLD. However, previous studies have focused on serum metabolites (9, 11–15), and to date, no studies have investigated bile metabolites.

We hypothesized that specific gallbladder bile metabolites may be altered in MASLD in relation to fibrosis severity. The aim of this study was to identify bile-derived metabolites associated with hepatic fibrosis by performing metabolomic profiling of gallbladder bile samples from biopsy-proven MASLD patients and healthy controls.

## Methods

### Study design

The study sample was obtained from a prospectively assembled cohort of patients at two hospitals in Korea. Written informed consent was obtained from all participants. This study received approval from the institutional review boards at each hospital (Nos. KBSMC 2019-08-007–031 and 2019-12-028-013).

### Characteristics of subjects

This cohort study included adults 20 years and older who had undergone cholecystectomy for benign gallbladder disease. The majority of the cases underwent cholecystectomy due to gallbladder polyps as an elective operation. During the surgery, bile samples were collected from the gallbladders, and liver biopsies were conducted in segment IV of the gallbladder fossa utilizing laparoscopic scissors. The diagnosis of MASLD was based on histological evidence of hepatic steatosis, coupled with the exclusion of secondary causes for the presence of hepatic steatosis. To exclude participants with acute cholecystitis, those meeting the criteria for moderate-to-severe acute cholecystitis (grade II or III) according to the Tokyo Guidelines 2018 were excluded. In addition, to rule out liver disease from other causes, the following exclusion criteria were applied (16): significant elevation in the levels of liver enzymes aspartate aminotransferase (AST) and alanine aminotransferase (ALT) exceeding five times the upper limit of normal (ULN), total bilirubin exceeding three times the ULN, or platelet count <50 × 10^3^/mm^3^; alcohol intake ≥210 g/week for men and ≥140 g/week for women (17); viral hepatitis (hepatitis B or C); and past or current use of medications known to cause MASLD, including valproate, amiodarone, methotrexate, tamoxifen, or corticosteroids.

### Histopathology

An experienced pathologist evaluated the liver specimens, which were surgically resected, fixed in formalin, and stained with hematoxylin and eosin (H&E), and reported the non-alcoholic fatty liver disease activity score (NAS; 0–8) and fibrosis stage (1–4) based on the guidelines of the MASH Clinical Research Network (18). MASH was defined as a NAS of 5 or higher, and steatosis was defined as a NAS of 1–4.

### Metabolomic analyses

Bile was obtained from the gallbladder immediately after cholecystectomy in the operating room. The collected bile samples were immediately stored at −72°C. Metabolomic profiling of gallbladder bile was performed using Liquid Chromatography–Quadrupole Time-of-Flight Mass Spectrometry (LC-QTOF-MS) with methanol-based extraction. Peak alignment and annotation were conducted using MetaboAnalyst and CAMERA, and putative metabolites were matched to the Human Metabolome Database (HMDB). The details of sample preparation and spectra processing are provided in the **Supplementary Methods**.

### Cell culture and treatment

HT-29 cells were cultured under standard conditions and treated with various concentrations of allyl nonanoate. Detailed culture media, seeding densities, and treatment protocols are described in the **Supplementary Methods**.

### Quantitative real-time polymerase chain reaction and Western blotting analysis

Total RNA was extracted using TRIzol reagent, and cDNA was synthesized using a commercial kit. The gene expression levels of GLP-1 and GIP were assessed by quantitative real-time polymerase chain reaction (qRT-PCR) using SYBR Green chemistry and normalized to β-actin. Detailed protocols are provided in the **Supplementary Methods**. Western blotting was performed on lysates from treated cells using antibodies against FN1, COL1A1, and GAPDH. Protein levels were quantified following Sodium Dodecyl Sulfate–Polyacrylamide Gel Electrophoresis (SDS-PAGE) and transferred to Polyvinylidene Fluoride (PVDF) membranes. Signals were detected using enhanced chemiluminescence and analyzed using the Image Lab software. The detailed procedures and antibody information are provided in the **Supplementary Methods**.

### Bulk RNA sequencing

Bulk RNA sequencing was performed on allyl nonanoate-treated HT-29 cells using the Illumina NovaSeq 6000 platform (paired-end 150 bp). Transcript-level data were processed through a standard FASTQ pipeline. Additional technical details are described in the **Supplementary Methods**. Transcriptome analysis of HT-29 cells was performed to evaluate the changes in gene expression induced by allyl nonanoate and to identify associated pathways. Gene set enrichment analysis (GSEA) was conducted using the GSEA software v.4.3.2 (University of California San Diego, CA, USA; and Broad Institute, Cambridge, MA, USA). The hallmark gene set collection was used to identify enriched pathways. Pathways with a false discovery rate (FDR) q-value < 0.25 were considered significantly enriched. The raw sequence data reported in this paper have been deposited in the Genome Sequence Archive (GSA-Human: HRA012170) in the National Genomics Data Center, China National Center for Bioinformation / Beijing Institute of Genomics, Chinese Academy of Sciences, which are publicly accessible at https://ngdc.cncb.ac.cn/gsa-human.

### GLP-1 and GIP induction in GPR119-overexpressing HT-29 cells

To assess the functional role of GPR119 in mediating the effects of allyl nonanoate, HT-29 cells were transduced with a GPR119 expression construct and treated with allyl nonanoate at various concentrations. GLP-1 and GIP mRNA expression levels were measured using qRT-PCR, confirming GPR119-dependent transcriptional activation. The experimental details are provided in the **Supplementary Methods**.

### GPCR screening assay

To evaluate the interaction of allyl nonanoate with G protein-coupled receptors (GPCRs), a comprehensive screening was conducted using the gpcrMAX panel provided by Eurofins DiscoverX (Fremont, CA, USA) through Koma Biotech (Seoul, Republic of Korea). The assay encompassed 409 human GPCR targets, utilizing the PathHunter^®^ β-Arrestin recruitment technology based on enzyme fragment complementation (EFC). Allyl nonanoate was tested at a concentration of 10 μM, and receptor activation or inhibition was quantified by measuring chemiluminescent signals using a PerkinElmer Envision™ plate reader. Data analysis was performed using the CBIS software, with results expressed as a percentage of the maximal response elicited by known reference agonists for each receptor.

### Statistical analysis

Statistical analyses for clinical data were conducted using the R software (v.4.0.4). Two-sample t-test or Wilcoxon rank sum test for continuous variables, and chi-squared test or Fisher’s exact test for categorical variables were performed to examine statistical differences between the experimental groups. For comparison between the three groups, analysis of variance or the Kruskal–Wallis test was used for continuous variables, and the chi-square or Fisher’s exact test was used for categorical variables. For pairwise comparisons, *post-hoc* analysis with Bonferroni correction was used. A p-value <0.05 was considered significant. Chemometric and statistical analyses for metabolomics data were performed using MetaboAnalyst 5.0. Principal component analysis (PCA) was conducted to visualize clustering patterns and identify group differences. Metabolite intensities were normalized using the median, and a 40% interquartile range filter was applied to remove low-variance features. Univariate analysis was performed to determine fold changes and adjusted p-values (FDR correction) between groups. For significant features, a threshold of fold change >2 and adjusted p-value <0.1 was used to identify differentially expressed metabolites. Heatmaps and volcano plots were generated to visualize metabolite profiles. For *in vitro* experiments, statistical analyses were performed using GraphPad Prism (version 9.0). Differences between groups were evaluated using one-way ANOVA followed by Tukey’s *post-hoc* test for multiple comparisons. A p-value <0.05 was considered statistically significant.

## Results

### Baseline characteristics of controls and subjects with fibrosis

The clinical characteristics of the study population are summarized in **Table 1**, encompassing a total of 68 subjects. The cohort was subdivided into three groups based on the histological severity: healthy controls (n = 19), MASLD patients with fibrosis stage 0–1 (n = 35), and MASLD patients with fibrosis stage 2–3 (n = 14). MASLD patients with mild fibrosis (stage 0–1) exhibited significantly higher levels of body mass index (BMI), plasma ALT, and AST compared to the control group (p = 0.01, p = 0.006, and p = 0.006, respectively). In MASLD patients with fibrosis stage 2–3, there was a higher prevalence of type 2 diabetes mellitus and hypertension, as well as higher serum levels of ALT, AST, and triglycerides, compared to MASLD patients with fibrosis stage 0–1.

**Table 1:** Patient characteristics with mild and significant fibrosis.

Parameter	Control (n = 19)	MASLD with fibrosis stage (0–1) (n = 35)	MASLD with fibrosis stage (2–3) (n = 14)	p-Value
Age (years)	46.5 ± 9.6	43.1 ± 12.4	52.6 ± 9.5	0.025
Gender (F/M)	9/10	20/15	9/5	0.570
BMI (kg/m^2^)	23.9 ± 3.9	27.4 ± 3.8	27.0 ± 4.3	0.013
Hypertension, n (%)	4 (21.1%)	9 (25.7%)	8 (57.1%)	0.075
Diabetes, n (%)	3 (15.8%)	1 (2.9%)	7 (50.0%)	<0.001
ALT (U/L)	16 (12.0, 21.5)	21 (17.0, 47.5)	27 (21.5, 57.8)	0.003
AST (U/L)	18 (15.5, 21.0)	21 (18.0, 29.0)	29 (22.0, 46.3)	0.002
Cholesterol (mg/dL)	176.6 ± 39.5	190.4 ± 33.4	162.6 ± 41.4	0.080
Triglyceride (mg/dL)	120 (103.0, 151.0)	155 (101.8, 195.0)	202.5 (148.0, 283.3)	0.071

Data are mean ± standard deviation, n (%), or median (IQR).

BMI, body mass index; ALT, alanine aminotransferase; AST, aspartate aminotransferase; IQR, interquartile range.

Mild fibrosis = fibrosis stage 0–1; significant fibrosis = fibrosis stage 1–2.

### Multivariate logistic regression of allyl nonanoate in hepatic fibrosis

We performed a multivariate stepwise logistic regression analysis to adjust for potential confounding variables, including age, sex, BMI, and relevant clinical parameters. As shown in **Table 2**, allyl nonanoate remained a statistically significant and independent risk factor for hepatic fibrosis across all models. In Model 1, after adjusting for age and sex, allyl nonanoate showed a significant association with hepatic fibrosis (p = 0.002, OR = 1.001). This association persisted after further adjustment for BMI, glucose, and cholesterol in Model 2 (p = 0.006, OR = 1.001) and remained significant even in the fully adjusted model, including bilirubin, AST, ALT, and gamma-glutamyl transferase (GGT) (p = 0.010, OR = 1.001). These results indicate that the association between allyl nonanoate levels and hepatic fibrosis is independent of metabolic and hepatic function parameters, supporting its potential role as an independent biomarker related to hepatic fibrosis in MASLD.

**Table 2:** Multivariate stepwise logistic regression analysis of allyl nonanoate and clinical parameters for hepatic fibrosis.

	Model 1		Model 2		Model 3	
	*p-Value	Exp(B)	*p-Value	Exp(B)	*p-Value	Exp(B)
Allyl nonanoate	0.002	1.001	0.006	1.001	0.010	1.001
Sex	0.275	2.735	0.302	3.384	0.694	2.080
Age (years)	0.049	1.094	0.154	1.073	0.236	1.087
BMI (kg/m^2^)			0.657	0.949	0.323	0.838
Glucose (mg/dL)			0.686	1.033	0.345	1.042
Cholesterol (mg/dL)			0.302	0.985	0.585	0.989
Bilirubin (mg/dL)					0.266	0.030
AST (U/U)					0.121	1.355
ALT (U/L)					0.448	0.928
GGT (U/L)					0.132	0.978

Model 1: adjusted for sex and age. Model 2: further adjusted for body mass index (BMI), glucose, and cholesterol. Model 3: fully adjusted for bilirubin, aspartate aminotransferase (AST), alanine aminotransferase (ALT), and gamma-glutamyl transferase (GGT). Exp(B) indicates the odds ratio (OR). All continuous variables were analyzed as continuous values.

*p-Values were calculated using the Wald test.

### Bile metabolite profiles in MASLD were distinct compared with healthy controls

**Figure 1:**
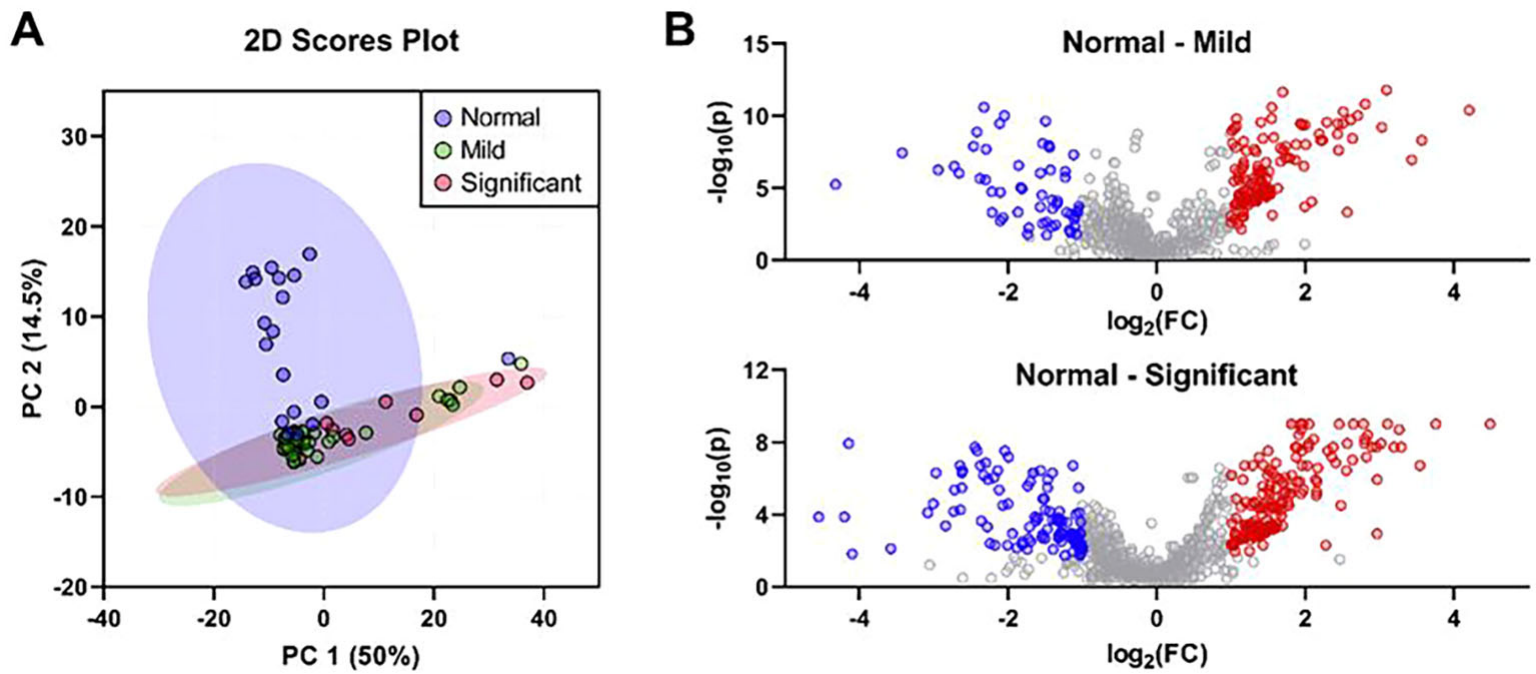
The differentially expressed bile metabolites were identified based on the histological severity of fibrosis. **(A)** Principal component analysis of bile metabolic profiling in controls (n = 19), mild fibrosis patients (n = 35), and significant fibrosis patients (n = 14). **(B)** Volcano plots of significantly different bile metabolites in mild (top) and significant fibrosis patients (bottom). Metabolites highlighted as red dots represent those significantly increased in patients with mild or significant fibrosis, while blue dots indicate those significantly decreased compared to the controls (fold change >2 and an adjusted p-value <0.05).

**Figure 2:**
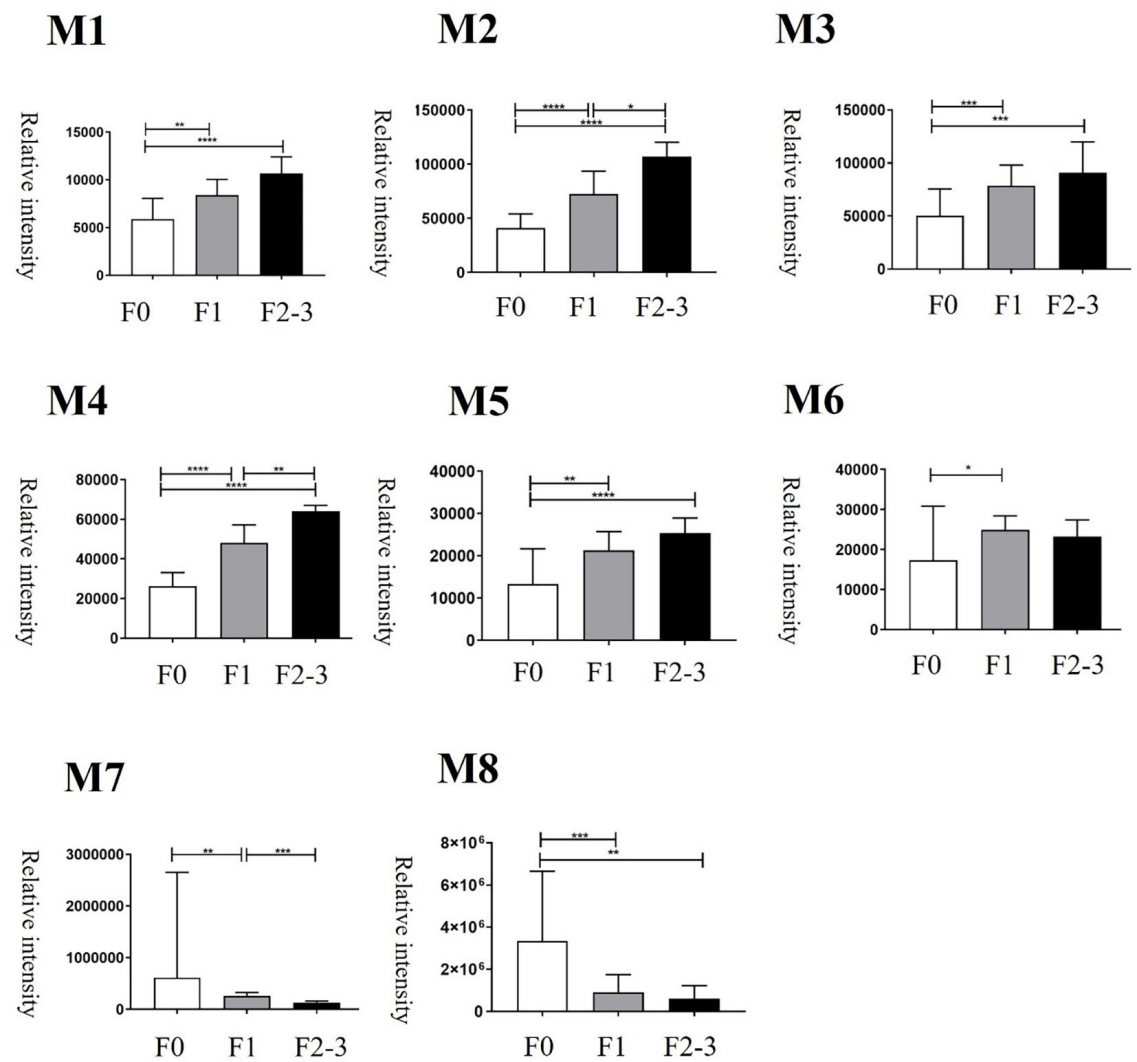
Changes of bile metabolites at different histologic severities of fibrosis. F0 = fibrosis grade 0, F1 = fibrosis grade 1, F2–3 = fibrosis grade 2–3. *p < 0.05, **p < 0.01, ***p < 0.001, and ****p < 0.0001.

PCA of bile metabolites revealed clear differences between healthy controls and MASLD patients (**Figure 1A**). The first two components (PC1 and PC2) accounted for 64.5% of the total variance (PC1, 50%; PC2, 14.5%). Significant differences were observed between healthy controls and MASLD patients. Volcano plot analysis identified 139 and 173 upregulated metabolites in the mild and significant fibrosis groups, respectively, compared with healthy controls, as well as 58 and 117 downregulated metabolites in the mild and significant fibrosis groups, respectively (fold change >2, adjusted p < 0.1; **Figure 1B**). Notably, eight bile metabolites not only elevated in MASLD patients compared with healthy controls but also increased progressively with fibrosis severity (**Figure 2**).

### Bile allyl nonanoate increased in MASLD patients and progressively with fibrosis severity

**Figure 3:**
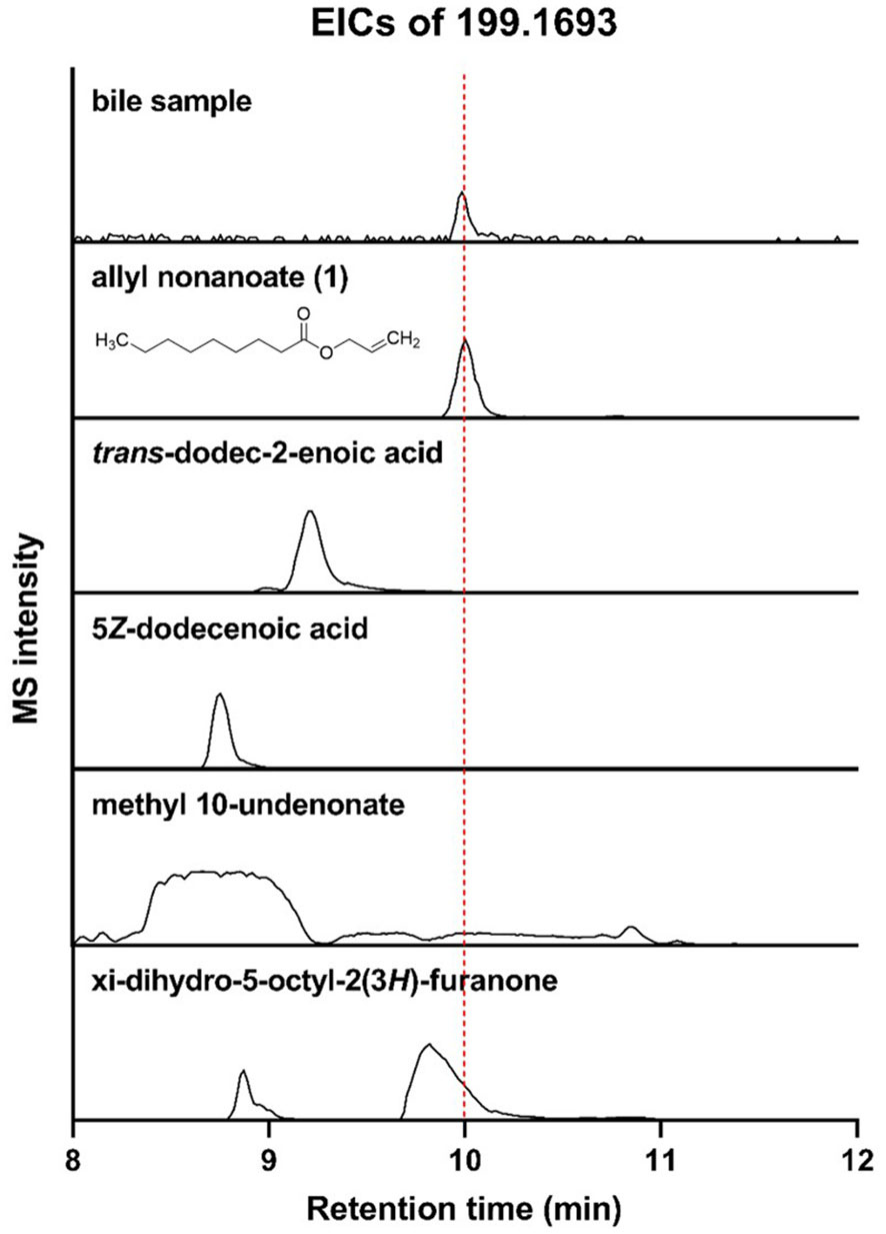
Liquid Chromatography–High-Resolution Quadrupole Time-of-Flight Mass Spectrometry (LC-HR-QTOF-MS) analysis of bile sample and five candidates for M5 using extracted ion chromatograms (EICs; *m*/*z* 199.1693).

Among the metabolites that were increased or decreased in MASLD patients compared with healthy controls, eight bile metabolites (M1–M8) showing a dose-dependent pattern according to fibrosis stage were finally selected (**Figure 2**). To precisely identify these eight bile metabolites, the HMDB was queried, which yielded a total of 19 putative metabolite candidates (**Supplementary Table 1**). Authentic standards for the candidate metabolites were obtained when available, and the mass-to-charge ratios (*m/z*) of the eight bile metabolites were re-examined for confirmation. As a result, only M5 could be unequivocally matched to its corresponding reference standard, whereas the others did not match any of the tested authentic compounds. Metabolite M5, with an *m/z* of 199.1693, fell within the specified mass tolerance range. Its structural identity was validated through direct comparison with an authentic allyl nonanoate standard, with the High-Resolution Mass Spectrometry (HRMS) retention time of M5 showing an exact match to that of the reference compound (**Table 3**, **Figure 3**). Furthermore, targeted Tandem Mass Spectrometry (MS/MS) analysis revealed a fragmentation pattern consistent with the standard, thereby confirming M5 as allyl nonanoate (**Supplementary Table 2**).

**Table 3:** Comparison of retention times between the metabolite with *m/z* 199.1693 in bile samples and authentic standards of the five candidates.

Metabolite	Retention time (min)
Bile samples	9.988
Allyl nonanoate	10.004
*trans*-Dodec-2-enoic acid	9.197
5*Z*-Dodecenoic acid	8.747
Methyl 10-undecenoate	8.638
xi-Dihydro-5-octyl-2(3*H*)-furanone	9.810

### Allyl nonanoate is associated with pathways related to cell death

**Figure 4:**
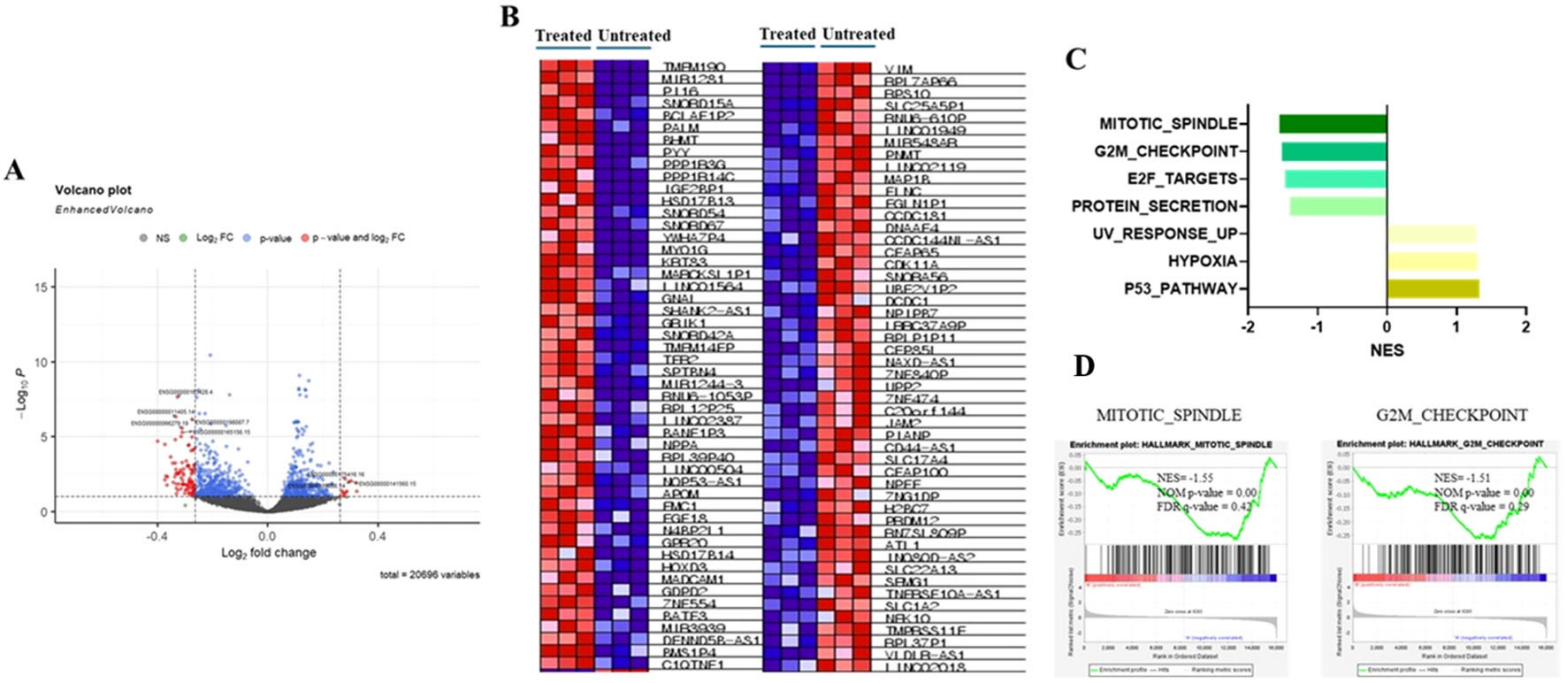
Changes in mRNA expression in HT-29 cell lines after allyl nonanoate treatment. **(A)** Volcano plot of genes differentially expressed in allyl nonanoate-treated HT-29 cells vs. untreated cells. **(B**–**D)** Gene set enrichment analysis (GSEA) results comparing allyl nonanoate-treated cells with controls. **(B)** Heatmap of the top 50 marker genes in the comparison of allyl nonanoate (left column) vs. control (right column). **(C)** Results of GSEA hallmark analysis showing enriched gene sets. **(D)** Enrichment plots for two datasets with an absolute normalized enrichment score (|NES|) ≥1.5.

To investigate the potential effects of allyl nonanoate (secreted into bile, which is synthesized in the liver and secreted into the intestine) on intestinal epithelial cells, HT-29 cells were treated with 1 μM/mL allyl nonanoate for 24 hours, followed by bulk RNA sequencing. Differentially expressed gene (DEG) analysis between the treatment and control groups (**Figures 4A, B**, **Supplementary Figure**) revealed that allyl nonanoate treatment increased the expression of CLTB and FN3KRP, which are functionally linked to protein fructosylation, glycation, and GLUT4-related pathways, while decreasing the expression of PIK3C2A, a gene that directly enhances insulin signaling through the regulation of phosphoinositide metabolism. Furthermore, the expression of ASPM, which promotes cell division and proliferation while inhibiting apoptosis, was significantly suppressed. Kyoto Encyclopedia of Genes and Genomes (KEGG) pathway analysis based on these upregulated and downregulated genes showed that allyl nonanoate treatment inhibited pathways related to cell growth and division while upregulating hypoxia-related pathways (**Figure 4C**).

### Allyl nonanoate regulates GLP-1 and GIP expression through GPR119

**Figure 5:**
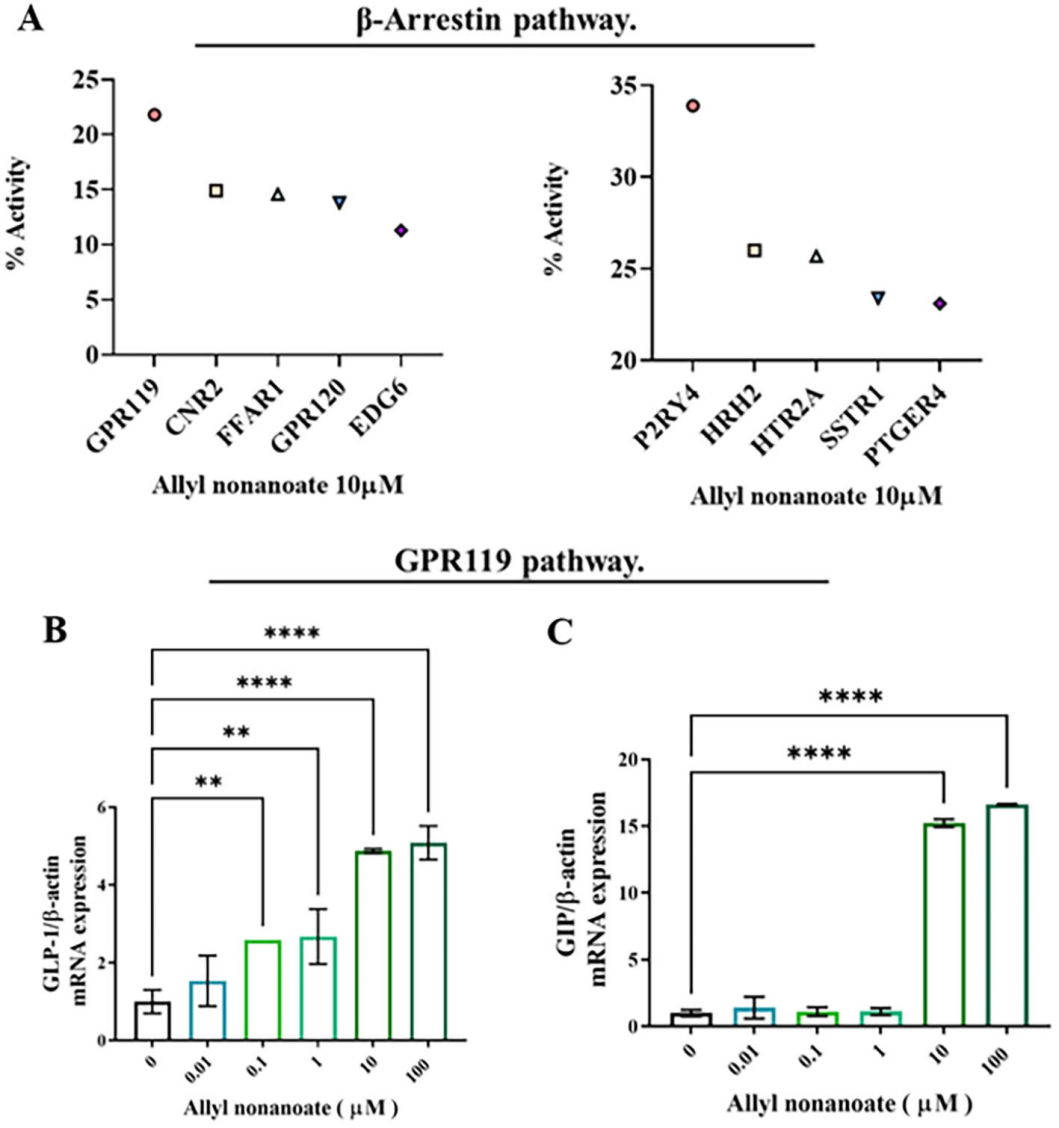
Induction of incretin hormone expression via GPR119 activation following allyl nonanoate treatment. **(A)** Results of a G protein-coupled receptor (GPCR) screening assay evaluating agonist and antagonist activities in the GPCR β-arrestin pathway. **(B, C)** Quantitative RT-PCR analysis of GLP-1 **(B)** and GIP **(C)** gene expression in HT-29 cells treated with allyl nonanoate, demonstrating activation of the GPR119 signaling pathway. *p < 0.05, **p < 0.01, ****p < 0.0001.

To elucidate the receptor and ligand pathways through which bile-secreted allyl nonanoate influences cell survival, death, GLUT4 regulation, and protein glycosylation, a GPCR array analysis was performed. This assay utilized EFC technology based on β-arrestin recruitment, and chemiluminescent signals were measured following treatment with 10 μM allyl nonanoate to quantify the activation or inhibition of each receptor. Screening revealed that GPR119 exhibited the highest agonist activity, with an efficacy of 21.8%, while in antagonist mode, P2RY4 showed the greatest inhibition at 33.9% (**Figure 5A**). In intestinal epithelial cells, allyl nonanoate treatment significantly increased GLP-1 and GIP mRNA expression (**Figure 5B**).

## Discussion

This study demonstrated that gallbladder bile metabolites differ according to hepatic fibrosis severity in MASLD patients. By directly analyzing bile from the gallbladder, we identified allyl nonanoate as a fibrosis-associated metabolite that increases in a stepwise pattern with fibrosis progression. This approach offers a novel perspective distinct from previous serum- or feces-based studies and highlights the value of gallbladder bile in uncovering liver-specific metabolic signatures relevant to MASLD.

Allyl nonanoate demonstrated consistent antifibrotic potential across multiple experimental systems. In hepatic stellate cells, it downregulated fibrosis-related genes; in intestinal epithelial cells, it modulated transcriptional programs related to cell cycle arrest; and in a GPCR screening platform, it activated GPR119, promoting the expression of incretin hormones known to attenuate fibrosis. Together, these findings suggest that allyl nonanoate exerts multifaceted antifibrotic effects through both the direct suppression of fibrogenic pathways and indirect hormonal signaling mechanisms.

Transcriptomic analysis of HT-29 cells revealed the downregulation of the G2/M checkpoint and the mitotic spindle pathway, consistent with the anti-proliferative effect observed in epithelial fibrotic injury models. Previous studies have demonstrated that G2/M arrest following epithelial injury can contribute to maladaptive repair and fibrogenesis, whereas the resolution of this arrest promotes normal tissue regeneration. For example, the circular RNA circBNC2 has been shown to prevent fibrotic remodeling by inhibiting G2/M arrest in kidney and liver epithelial cells (19). Additionally, studies on hepatic stellate cells have shown that inflammatory mediators can induce G2/M phase arrest, influencing cell proliferation and motility during liver fibrosis (20). Although these pathways did not meet stringent thresholds for statistical significance (FDR < 0.25), their biological plausibility and consistency with known fibrosis-related mechanisms suggest a potential link between allyl nonanoate and epithelial cell cycle dynamics. These findings should be regarded as preliminary, and further studies are warranted to determine whether such transcriptomic responses translate into functional outcomes *in vivo*.

In a GPCR screening assay, GPR119 exhibited the strongest agonist response to allyl nonanoate and was selected for further validation given its established role in incretin regulation. GLP-1 and GIP, two major incretin hormones, are increasingly recognized for their potential antifibrotic roles in the liver (21–23). In our study, allyl nonanoate treatment led to a significant increase in GLP-1 and GIP mRNA expression in GPR119-overexpressing HT-29 cells, supporting its agonistic effect. Interestingly, comparable or even greater responses were observed in parental HT-29 cells at higher concentrations, suggesting that GPR119-independent or off-target mechanisms may also contribute to the observed effects.

Allyl nonanoate is an exogenous metabolite widely used as a synthetic flavor additive in processed foods enriched with simple sugars, particularly those flavored with pineapple or tropical fruit (24). Given the well-established association between MASLD and fructose consumption, it is plausible that elevated levels of allyl nonanoate may, at least in part, reflect increased dietary exposure to industrially derived fructose. Although it is not endogenously synthesized, allyl nonanoate can enter the body through dietary intake or environmental exposure.

Nevertheless, in our study, bile samples from patients with advanced fibrosis consistently showed higher concentrations of allyl nonanoate compared with controls recruited under the same clinical conditions. This pattern is unlikely to be explained solely by exogenous exposure and may instead suggest altered absorption, metabolism, or excretion processes in the setting of fibrosis. Such findings raise the possibility that allyl nonanoate or its metabolites interact with the liver–gut axis, perhaps through microbiota-mediated transformation or impaired hepatic clearance.

Taken together, these observations highlight both the potential and limitations of interpreting allyl nonanoate as a biomarker. While it may partly reflect dietary exposure, its selective enrichment in fibrotic bile suggests an involvement in disease-associated metabolic alterations. Further studies, including diet-controlled investigations, validation in independent cohorts, and mechanistic experiments in animal models, will be necessary to clarify whether allyl nonanoate functions as a fibrosis-specific biomarker or an indirect indicator of dietary intake.

The limitations of this study are as follows. First, the metabolite analysis performed in this study was based on an untargeted approach. In the future, applying a targeted metabolite analysis focused on specific pathways may enable the identification of a greater number of metabolites and provide a deeper understanding of the underlying mechanisms. Second, this study primarily investigated the effects and mechanisms of allyl nonanoate excreted in bile on intestinal epithelial cells. However, allyl nonanoate present in bile may also exert direct effects on various cell types within the liver. Therefore, further studies are needed to examine its direct impact on major hepatic cell populations. Third, allyl nonanoate is generally presumed to be an exogenous compound and is widely used as a flavoring agent to impart various fruit aromas, often in combination with industrial fructose. The increased levels of allyl nonanoate in bile may be associated with higher consumption of industrial fructose or fast food in MASLD patients. However, this study lacked a detailed assessment of the specific dietary patterns of the MASLD cohort. Fourth, this study focused on MASLD-related fibrosis, and the findings should not be generalized to other etiologies of liver fibrosis (**Supplementary Figure 4**). Future studies with larger cohorts will be important to validate and extend these fibrosis- and metabolism-related findings.

In this study, we comprehensively profiled bile metabolites in MASLD patients and demonstrated distinct differences compared with healthy controls. Importantly, several metabolites were not only associated with the presence of MASLD but also correlated with the severity of hepatic fibrosis, directly addressing our research question regarding potential metabolic markers of disease progression. These findings highlight the clinical relevance of bile metabolites as promising biomarkers for the early detection and stratification of MASLD and may also provide novel insights into therapeutic targets that warrant further investigation.

## Data Availability

The datasets presented in this study can be found in online repositories. The names of the repository/repositories and accession number(s) can be found in the article/**Supplementary Material**.
