# Supplementary material for: Allyl nonanoate as a novel bile-derived biomarker in metabolic dysfunction-associated steatotic liver disease
Source: Front Endocrinol (Lausanne). 2025 Oct 28;16:1707492. doi: 10.3389/fendo.2025.1707492 (PMC12602178; doi:10.3389/fendo.2025.1707492)
Supplement: Supplementary file 1 [file DataSheet1.docx]

**Supplementary Methods**

**Metabolomic Analysis of Bile Samples**

*Sample Extraction*

Bile samples (50 μL) were extracted using methanol (450 μL), vortexed, and centrifuged at 13,800 rpm for 5 min. The resulting supernatant (100 μL) was diluted with 900 μL methanol, and 1000 μL was transferred to LC-MS vials. Standard compounds (allyl nonanoate, methyl 10-undecenoate, xi-dihydro-5-octyl-2(3H)-furanone, 5Z-dodecenoic acid, and trans-dodec-2-enoic acid) were diluted to 100 μg/mL and analyzed in parallel.

*Instrumentation and Chromatographic Conditions*

High-resolution electrospray ionization mass spectrometry (HRESIMS) was performed using an Agilent 6545B Q-TOF MS system coupled to an Agilent 1260 Infinity II UPLC. Separation was achieved on an Eclipse Plus C18 column (2.1 mm × 50 mm, 1.8 μm) at a flow rate of 0.3 mL/min. The mobile phase gradient was as follows: 0–10 min, 10–100% MeCN in water with 0.1% formic acid; 10–12 min, 100% MeCN; 12–15 min, return to 10% MeCN.

*MS/MS Analysis*

Targeted MS/MS analysis used the same LC-QTOF-MS configuration. Data were acquired in positive ion mode under the following conditions: gas temperature 320 °C; gas flow 8 L/min; sheath gas temperature 350 °C; sheath gas flow 11 L/min; nebulizer pressure 35 psi; capillary voltage 3500 V; nozzle voltage 1000 V. Fragmentor voltage was 100 V, and CID energies of 10, 20, and 30 eV were applied. Acquisition rate was 5 spectra/s across an MS/MS range of 100–1700 m/z.

*Data Processing and Compound Identification*

Raw data were converted to mzML format using MSConvert (ProteoWizard) [1]. Centroided spectra were processed in MetaboAnalyst 5.0 using the centWave algorithm [2]. Peak annotation was performed with the CAMERA package [3], and molecular formulae and compound IDs were curated using the Human Metabolome Database (HMDB) [4].

**In Vitro Experiments**

*Cell Culture and Treatment*

The HT-29 human colon adenocarcinoma cell line was cultured in RPMI 1640 medium (Gibco, Thermo Fisher Scientific, Cat. No. 22400089) supplemented with 10% fetal bovine serum (FBS; Gibco, Cat. No. 1600044) and 1% penicillin-streptomycin (Welgene, Korea; Cat. No. LS202-02). Cells were seeded at a density of 3 × 10⁵ cells/well in 6-well plates (SPL Life Sciences, Korea; Cat. No. 30006). After 24 hours, the medium was replaced, and cells were treated with 1 μM allyl nonanoate (TCI, Japan; Cat. No. A2679). Samples were collected after 24 hours.

HepG2 human hepatocyte cells were cultured in Dulbecco’s Modified Eagle Medium (DMEM; Gibco, Thermo Fisher Scientific, Cat. No. 11885084) supplemented with 10% fetal bovine serum (FBS; Gibco, Cat. No. 1600044) and 1% penicillin-streptomycin (Welgene, Korea; Cat. No. LS202-02). Cells were seeded at a density of 5 × 10³ cells/well in 96-well plates (Thermo Fisher Scientific, USA; Cat. No. 167008 or 165305). After 24 h, cells were stimulated with 400 μM oleic acid (OA; Sigma-Aldrich, Germany; Cat. No. O1257) or palmitic acid (PA; Sigma-Aldrich, USA; Cat. No. P5585) and subsequently treated with allyl nonanoate (0.01, 0.1, 1, 10, or 100 μM; TCI, Japan; Cat. No. A2679) for an additional 24 h.

The LX-2 human hepatic stellate cell line was maintained in Dulbecco’s Modified Eagle Medium (DMEM; Gibco, Thermo Fisher Scientific, Cat. No. 11965092) supplemented with 2% FBS (Gibco, Cat. No. 1600044) and 1% penicillin-streptomycin (Welgene, Cat. No. LS202-02). Cells were seeded at a density of 3 × 10⁵ cells/well in 6-well plates (SPL Life Sciences, Korea; Cat. No. 30006). After 24 hours, cells were stimulated with 10 ng/mL transforming growth factor (TGF)-β (R&D Systems, USA; Cat. No. 204B) and then treated with allyl nonanoate (0.01, 0.1, 1, and 10 μM; TCI, Japan; Cat. No. A2679) for an additional 24 hours.

Detailed information on the cell lines used in this study is provided in Supplementary Table 2.

*RNA Extraction and qRT-PCR*

Cells were lysed using 1 mL of TRIzol reagent (Invitrogen, Cat. No. 15596018), followed by the addition of 200 μL of chloroform. After mixing and centrifugation at 12,000 rpm for 15 minutes at 4°C, the aqueous phase was transferred to a new tube, and RNA was precipitated with 500 μL of isopropanol. Samples were incubated at 4°C for 10 minutes and centrifuged at 12,000 rpm for 10 minutes. The resulting RNA pellet was washed with 75% ethanol, centrifuged at 8,500 rpm for 5 minutes, air-dried, and dissolved in DEPC-treated water. RNA concentration and purity were assessed using a NanoDrop spectrophotometer (Thermo Fisher Scientific) based on A260/280 ratio.

Complementary DNA (cDNA) was synthesized using the PrimeScript 1st Strand cDNA Synthesis Kit (Takara Bio, Cat. No. 9765) according to the manufacturer’s instructions. qRT-PCR was performed using the LightCycler 480 SYBR Green I Master Mix (Roche) on a LightCycler 480 II system. Gene expression was normalized to β-actin.

*Western blot analysis*

For protein extraction, radioimmunoprecipitation assay (RIPA) lysis buffer (Thermo Fisher Scientific, Rockford, IL, USA; R4100-010), supplemented with protease inhibitors (GENDEPOT, USA; P3100-001) and phosphatase inhibitors (GENDEPOT, USA; P3200-001) at a final concentration of 1:100, was used. After collecting the cells with cold PBS and pelleting by centrifugation, the supernatant was removed. The cells were then lysed by adding the lysis mixture and incubating on ice for 30 minutes. The supernatant containing the proteins was collected and stored at -80°C until further analysis. Protein concentrations were determined using the Pierce™ BCA Protein Assay Kit (Thermo Scientific, USA; 23225), following the manufacturer’s instructions.

The extracted proteins were separated by gel electrophoresis and transferred to PVDF membranes (Immobilon-P; Millipore, Billerica, MA, USA). The membranes were blocked with 5% skim milk solution for 1 hour and then incubated with primary antibodies against FN-1 (Abcam, Cambridge, MA, USA, ab2413), Col1A1 (Abcam, Cambridge, MA, USA, ab260043), and GAPDH (GeneTex, USA, GTX-100118). Subsequently, they were incubated with appropriate secondary antibodies. Protein bands were visualized using an image analyzer (Image Lab 3.0; Bio-Rad, Hercules, CA, USA) and quantified.

*Bulk RNA sequencing*

Total RNA was extracted from HT-29 cells treated with 1 μM allyl nonanoate (n=3) and untreated control cells (n=3). Library preparation was performed using standard protocols. DNA concentration was measured using the Qubit DNA HS Assay (Thermo Fisher Scientific) for quality control. Libraries were sequenced as paired-end 150 bp reads on the Illumina NovaSeq 6000 platform. Image data were processed with Real-Time Analysis (RTA) software, and BCL files were converted to FASTQ format using bcl2fastq2 or bcl-convert with default parameters. Transcriptome profiling was performed on a total of 6 samples to evaluate gene expression changes between treated and control groups.

*GPR119 Overexpression*

HT-29 cells were transduced with a human GPR119 expression lentiviral particle (Origene, Cat. No. RC216685L3V) according to the manufacturer’s protocol and incubated for 24 hours. Stable overexpression was confirmed by qRT-PCR and Western blotting using an anti-Myc antibody. Cells were then seeded into 6-well plates (3 × 10⁵ cells/well) and treated with allyl nonanoate at concentrations of 0.1, 1, and 10 μM for 24 hours. GLP-1 and GIP mRNA expression levels were measured using qRT-PCR. HT-29 cells were transduced with a human GPR119 expression lentiviral particle (Origene, Cat# RC216685L3V) according to the manufacturer’s protocol and incubated for 24 hours. Stable overexpression was confirmed by qRT-PCR and Western blotting using an anti-Myc antibody. Cells were then seeded into 6-well plates (3 × 10⁵ cells/well) and treated with allyl nonanoate at concentrations of 0.1, 1, and 10 μM for 24 hours. GLP-1 and GIP mRNA expression levels were measured using qRT-PCR.

*CCK8 cell viability test*

Cell proliferation of HepG2 cells was assessed using the Cell Counting Kit-8 (CCK-8; CK04, Dojindo Laboratories, Kumamoto, Japan). After lipotoxicity was induced by treatment with palmitic acid (PA), cells were treated with various concentrations of allyl nonanoate for 24 h. Subsequently, CCK-8 reagent was added and incubated for 1.5 h, and absorbance at 450 nm was measured using a microplate reader (iMark, Bio-Rad, Hercules, CA, USA).

*Nile red staining*

To evaluate the effect of allyl nonanoate on lipogenesis after oleic acid (OA) stimulation, cells were washed twice with DPBS (Welgene, Korea, Cat. No. LB 001-02), fixed with 4% paraformaldehyde for 30 min at room temperature, and stained with Nile Red (0.5 mg/mL in acetone). Fluorescence intensity was measured at 488/550 nm using a Victor3 microplate reader (PerkinElmer, Turku, Finland).

References

1. Chambers MC, Maclean B, Burke R, Amodei D, Ruderman DL, Neumann S, et al. A cross-platform toolkit for mass spectrometry and proteomics. Nature biotechnology. 2012;30:918-20.

2. Pang Z, Zhou G, Ewald J, Chang L, Hacariz O, Basu N, et al. Using MetaboAnalyst 5.0 for LC–HRMS spectra processing, multi-omics integration and covariate adjustment of global metabolomics data. Nature protocols. 2022;17:1735-61.

3. Kuhl C, Tautenhahn R, Bottcher C, Larson TR, Neumann S. CAMERA: an integrated strategy for compound spectra extraction and annotation of liquid chromatography/mass spectrometry data sets. Analytical chemistry. 2012;84:283-9.

4. Wishart DS, Guo A, Oler E, Wang F, Anjum A, Peters H, et al. HMDB 5.0: the human metabolome database for 2022. Nucleic acids research. 2022;50:D622-D31.
