# Supplementary material for: Allyl nonanoate as a novel bile-derived biomarker in metabolic dysfunction-associated steatotic liver disease
Source: Front Endocrinol (Lausanne). 2025 Oct 28;16:1707492. doi: 10.3389/fendo.2025.1707492 (PMC12602178; doi:10.3389/fendo.2025.1707492)
Supplement: Supplementary file 2 [file DataSheet2.docx]

**Supplementary Table 1. Putative bile metabolites**

|  | Compound name |
| --- | --- |
| M1 | 11´-Carboxy-gamma-tocotrienol |
|  | Colupulone |
| M2 | Ceramide |
| M3 | *N*-Stearoyl taurine |
| M4 | 6-Dehydrotestosterone glucuronide |
|  | Neryl rhamnosyl-glucoside |
|  | xi-Linalool 3-[rhamnosyl-(1→6)-glucoside] |
| M5 | 5*Z*-Dodecenoic acid |
|  | *trans*-Dodec-2-enoic acid |
|  | Methyl 10-undecenoate |
|  | Allyl nonanoate |
|  | xi-Dihydro-5-octyl-2(3*H*)-furanone |
| M6 | Emedastine |
|  | Arginyllysine |
|  | Lysylarginine |
|  | xi-7-hydroxyhexadecanedioic acid |
|  | xi-8-hydroxyhexadecanedioic acid |
| M7 | AS1-1 |
| M8 | *N*-docosahexaenolyl GABA |

**Supplementary Table 2. Comparative analysis of positive ion fragmentation patterns for bile sample and allyl nonanoate standard based on HRMS-MS/MS data.**

|  | Bile sample | Allyl nonanoate |
| --- | --- | --- |
| Ion fragmentation | 111.0826 | 111.1166 |
|  | 121.0997 | 121.1016 |
|  | 123.0801 | 123.1170 |
|  | 139.1087 | 139.1122 |
|  | 141.0894 | 141.1274 |
|  | 181.0834 | 181.1593 |
|  | 199.1697 | 199.1697 |

**Supplementary Table 3. Cell lines used in this study**

| Name | Supplier | Cell type |
| --- | --- | --- |
| LX-2 | Professor Geon Wook Kang (Department of Nuclear Medicine, Seoul National University, Seoul, South Korea) | hepatic stellate cells |
| HT-29 | Korean Cell Line Bank (KCLB, Seoul, Korea) | colorectal cancer cells |
| HepG2 | Korean Cell Line Bank (KCLB, Seoul, Korea) | Hepatocyte cells |

**Supplementary Table 4. Sequences of qRT-PCR human primers**

| Gene | Primer |
| --- | --- |
| β-ACTIN | Forward: 5’-AGG-AAG-GAA-GGC-TGG-AAG-AG-3’ |
|  | Reverse: 5’-AGA-GCT-ACG-AGC-TGC-CTG-AC-3’ |
| FN1 | Forward: 5’-CGG-TGG-CTG-TCA-GTC-AAA-G-3’ |
|  | Reverse: 5’-AAA-CCT-CGG-CTT-CCT-CCA-TAA-3’ |
| Col1A1 | Forward: 5’- GTG-CTA-AAG-GTG-CCA-ATG-GT-3’ |
|  | Reverse: 5’-ACC-AGG-TTC-ACC-GCT-GTT-AC-3’ |
| TGF-β | Forward: 5’- CCC-TGG-ACA-CCA-ACT-ATT-GC-3’ |
|  | Reverse: 5’- GTC-CTT-GCG-GAA-GTC-AAT-GT-3’ |
| Col1A2 | Forward: 5’-CCT-GGT-GCT-AAA-GGA-GAA-AGA-GG-3’ |
|  | Reverse: 5’-ATC-ACC-ACG-ACT-TCC-AGC-AGG-A-3’ |
| ITGB1 | Forward: 5’- GGA-TTC-TCC-AGA-AGG-TGG-TTT-CG-3’ |
|  | Reverse: 5’- TGC-CAC-CAA-GTT-TCC-CAT-CTC-C-3’ |
| ITGAV | Forward: 5’- AGG-AGA-AGG-TGC-CTA-CGA-AGC-T -3’ |
|  | Reverse: 5’- GCA-CAG-GAA-AGT-CTT-GCT-AAG-GC -3’ |
