# Supplementary material for: Allyl nonanoate as a novel bile-derived biomarker in metabolic dysfunction-associated steatotic liver disease
Source: Front Endocrinol (Lausanne). 2025 Oct 28;16:1707492. doi: 10.3389/fendo.2025.1707492 (PMC12602178; doi:10.3389/fendo.2025.1707492)
Supplement: Supplementary file 3 [file DataSheet3.docx]

**
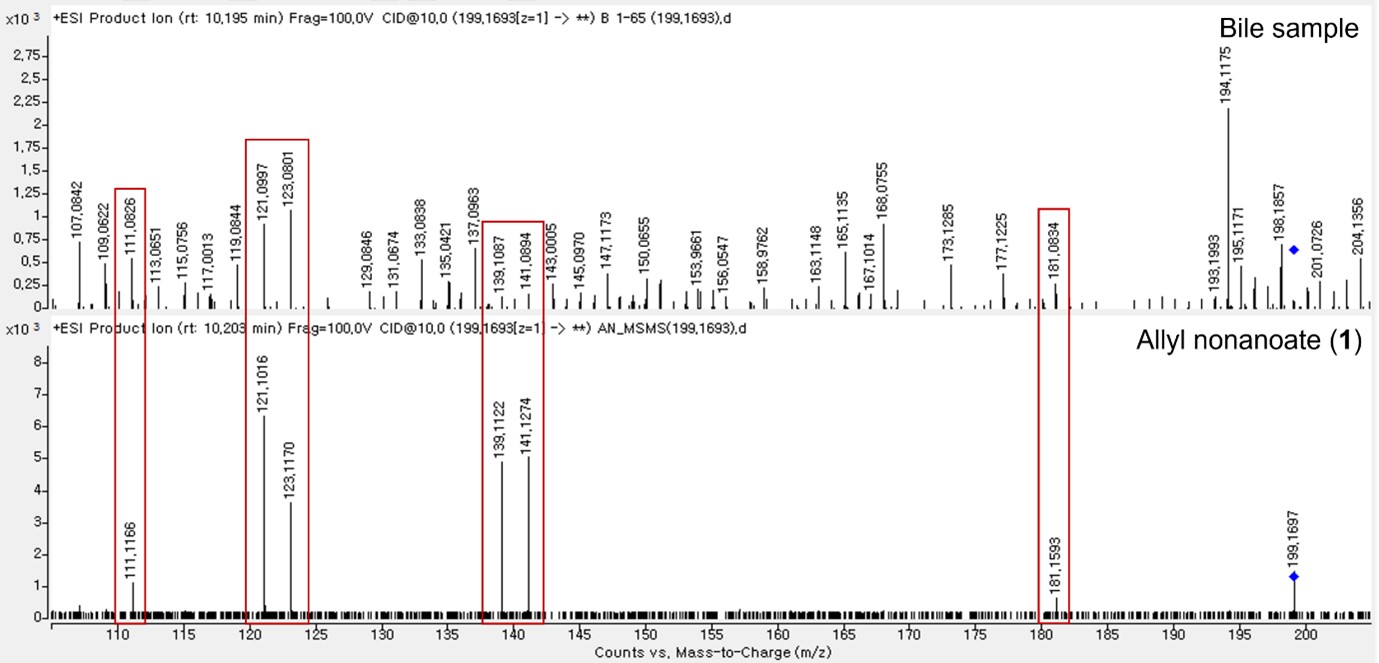
**

**Supplementary Figure 1.**

MS/MS spectra of allyl nonanoate in bile sample (top) and authentic standard (bottom).


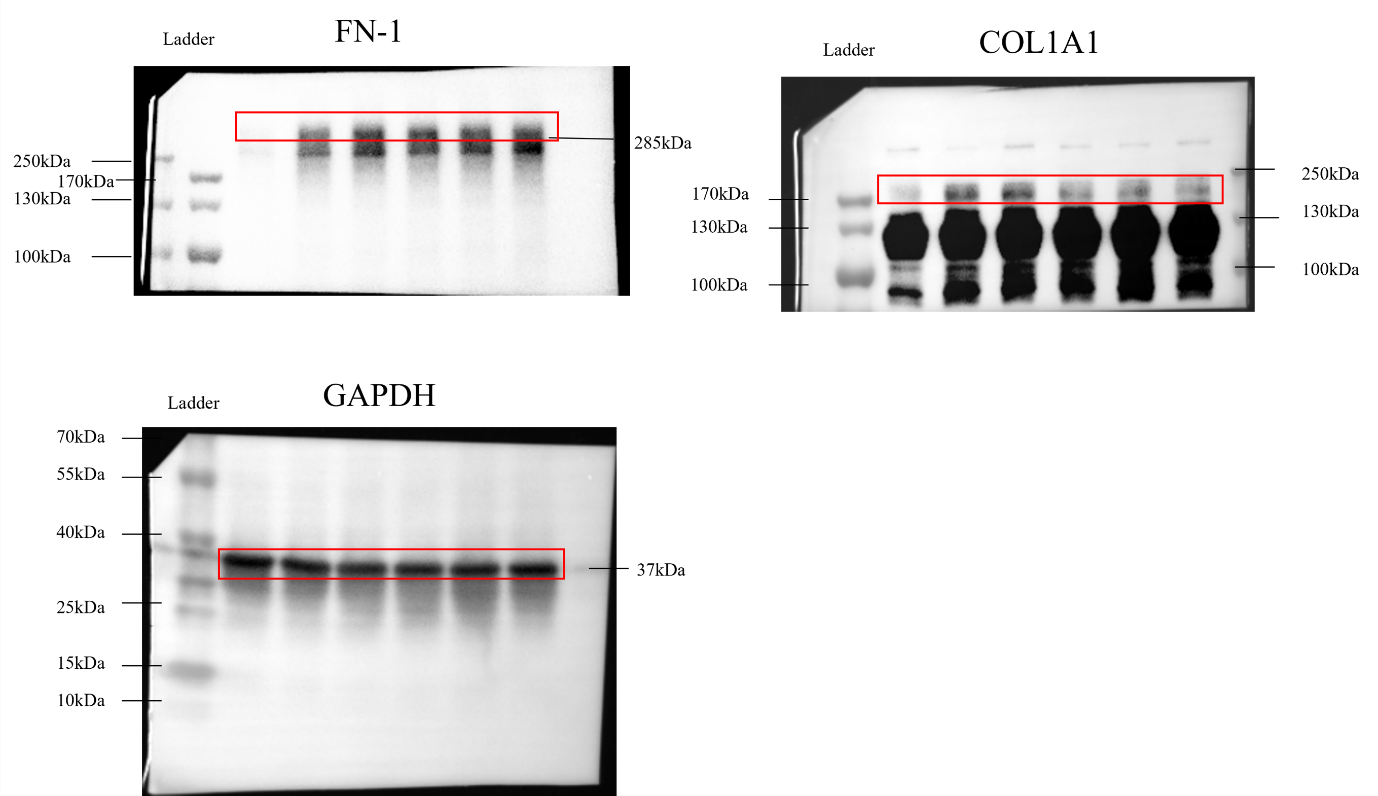


**Supplementary Figure 2.**

Full-length western blot images of FN-1, COL1A1, and GAPDH used in Figure 5. Molecular weight markers are indicated. Lane identities: 1=control, 2=TGF-β only, 3~6=TGF-β + allyl nonanoate (0.01, 0.1, 1, 10 μM). Cropped regions used in the main figure are indicated by red rectangles.

**
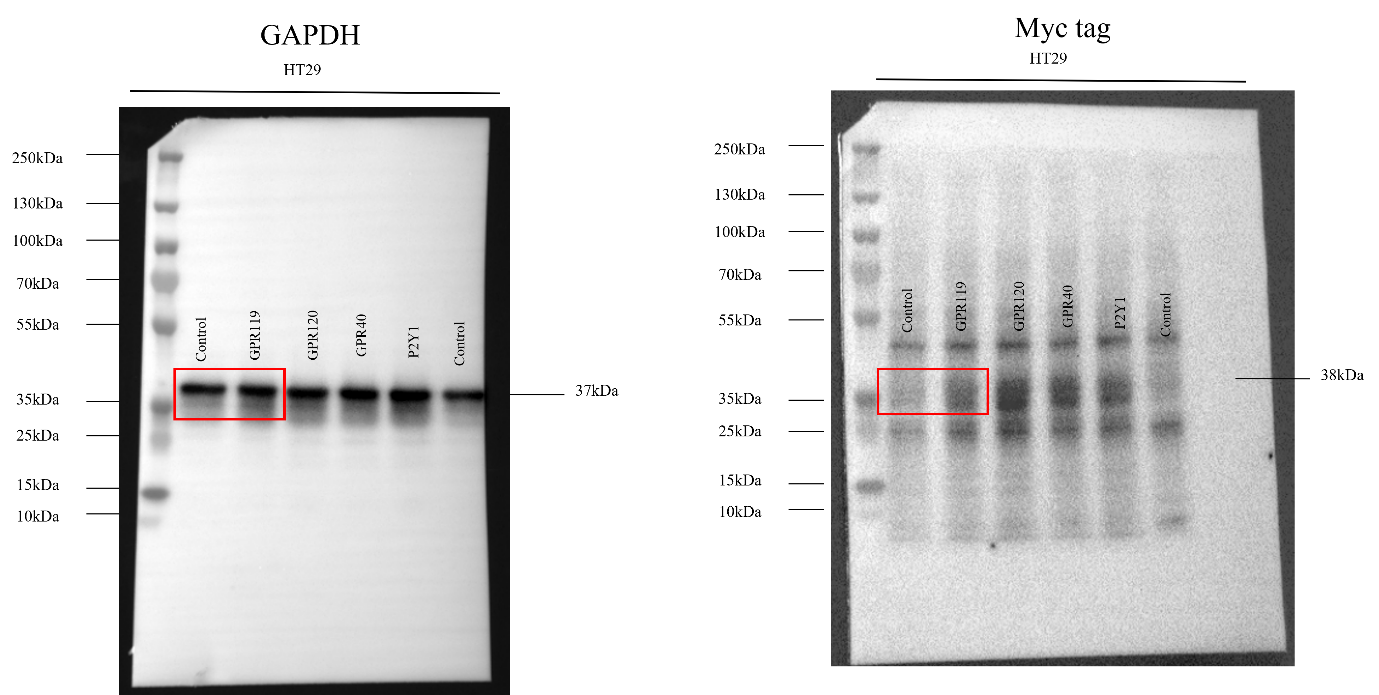
**

**Supplementary Figure 3.**

Full-length western blot images of Myc-tagged GPR119 and GAPDH used in Figure 6. Molecular weight markers are indicated. GPR119, GPR120, GPR40, and P2Y1 transfected HT29 cells as indicated. Cropped regions used in the main figure are indicated by red rectangles.

**Supplementary Figure 4.**

Expression levels of liver fibrosis markers (Col1a1, Col1a2, FN1, TGF-β, ITGB1, and ITGAV) were analyzed in hepatic stellate cells (LX2) using qRT-PCR.

**Supplementary Figure 5.**

HepG2 cells were exposed to oleic acid (400 μM) to induce lipogenesis or to palmitic acid (400 μM) to induce lipotoxicity, followed by treatment with allyl nonanoate at the indicated concentrations. Lipid accumulation was evaluated by Nile Red O staining, and cell viability was determined under lipotoxic conditions.


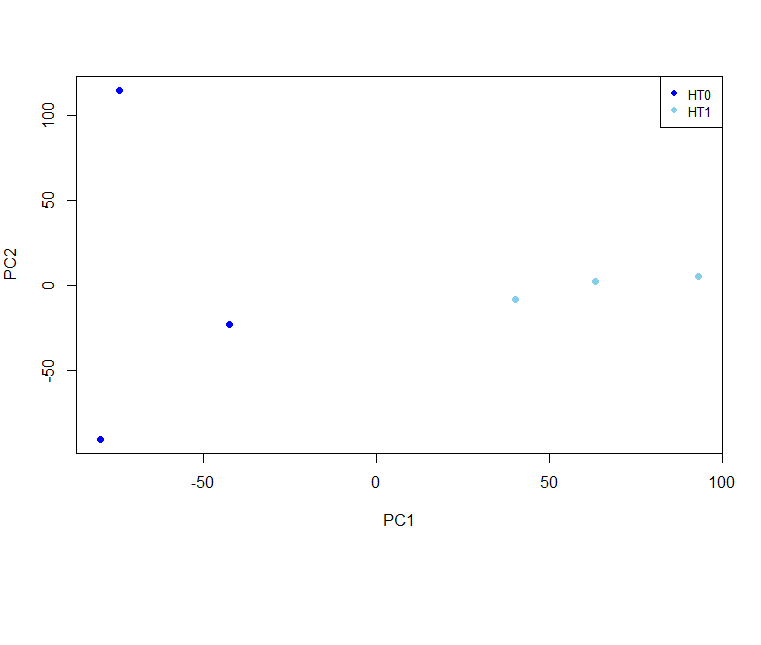


**Supplementary Figure 6.**

Principal Component Analysis confirming reproducibility of RNA-seq replicates.

PCA was conducted on RNA-seq data from HT29 cells (allyl nonanoate 1 μM, 24 h) and untreated controls. The analysis shows close clustering of replicates within each group, underscoring experimental reproducibility, and a clear separation between groups, reflecting treatment-specific gene expression changes.


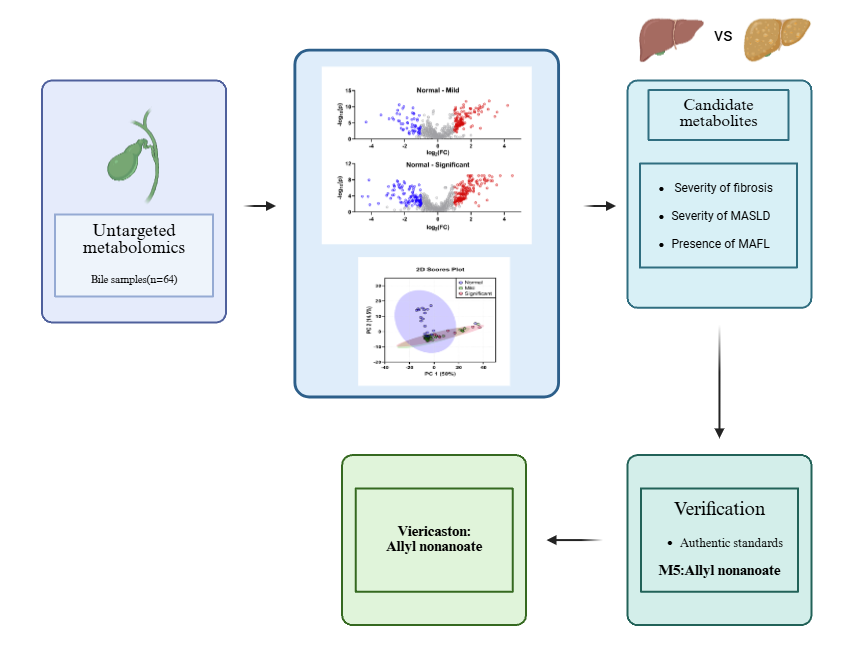


**Supplementary Figure 7.**

Overview of the experimental workflow illustrating the stepwise process from untargeted metabolomics (M1–M8) to the structural identification of metabolite M5 as allyl nonanoate.
